# Annealing-Controlled Recrystallization, Grain Growth, and Tensile Properties of Cold-Rolled L-605 Cobalt-Based Alloy

**DOI:** 10.3390/ma19183820

**Published:** 2026-09-08

**Authors:** Choi Seong-Woo, Chenglin Li

**Affiliations:** 1 LG Energy Solution, Seoul 07335, Republic of Korea; seongwoo1126@gmail.com; 2 School of Power and Mechanical Engineering, Wuhan University, Wuhan 430072, China; 3 Shenzhen Research Institute, Wuhan University, Shenzhen 518000, China

**Keywords:** L-605 alloy, Co–Cr–W–Ni alloy, biomedical alloy, annealing, static recrystallization, EBSD, KAM, grain growth, tensile properties

## Abstract

**Highlights:**

- Cold-rolled L-605 evolves from partial to near-full recrystallization at 800–950 °C.
- Fine recrystallized grains remain relatively stable at 1000 °C; fine second-phase particles may contribute to restricted grain-boundary migration.
- Annealing tunes yield strength from 1245 to 442–455 MPa and elongation from 12.6% to 80.8%.

**Abstract:**

This work investigated the effects of annealing temperature and holding time on recrystallization, grain growth, and tensile properties of cold-rolled biomedical Co–20Cr–15W–10Ni (L-605) alloy. The alloy was solution-treated at 1200 °C for 30 min, cold-rolled to 40% area reduction, and annealed at 800–950 °C for 15 min, 1000 °C for 5–60 min, or 1200 °C for 5–60 min. Annealing at 800–900 °C produced partially recrystallized microstructures; 950 °C yielded nearly full recrystallization with an average grain size of ~3.4 μm. At 1000 °C, the fully recrystallized microstructure retained ~5 μm fine grains even after 60 min, likely due to grain boundary migration inhibition by fine secondary-phase particles, whose composition and pinning effect remain unclarified. At 1200 °C, grains grew significantly to ~90 μm, attributed to higher grain boundary mobility and reduced particle pinning at elevated temperature. As annealing temperature rose from 800 °C to 950 °C, yield strength decreased from 1245 MPa to 800 MPa, and elongation increased from 12.6% to 45.9%. At 1200 °C, yield strength fell to 442–455 MPa and elongation reached 73.5–80.8%. The results confirm that the alloy’s strength–ductility performance is directly determined by microstructure evolution from retained cold rolling deformation substructures to fine recrystallized grains, and further to coarse recrystallized grains at higher temperatures.

## 1. Introduction

Co–Cr-based alloys are extensively applied in the biomedical field, attributed to their outstanding overall performance including biocompatibility, corrosion resistance, wear resistance and mechanical properties [1,2,3,4,5]. Among this class of alloys, Co–20Cr–15W–10Ni alloy, commercially designated as L-605, Haynes 25, and standardized as ASTM F90 [6], is particularly favored for cardiovascular stents and other load-bearing biomedical devices. For such application scenarios, both high strength and adequate ductility are mandatory to guarantee the mechanical reliability of the material during the forming process, in vivo implantation deployment, and long-term service [1,2,3,4,5,7,8]. Accordingly, precise regulation of the microstructure of this alloy is a critical prerequisite for optimizing its strength–ductility trade-off.

The mechanical behavior of L-605 alloy is strongly dependent on key microstructural characteristics, including grain size, recrystallization fraction, deformation-induced substructures, secondary-phase particles and phase stability. Cold deformation introduces orientation gradients and stores deformation energy within the alloy, while subsequent annealing treatment triggers recovery, static recrystallization (SRX) and grain growth in sequence [8,9]. At relatively low annealing temperatures, recovery can alleviate local lattice distortion without completely eliminating the deformed microstructure. As the annealing temperature or holding time increases, newly recrystallized grains nucleate and grow, gradually replacing the deformed matrix.

The existing literature has systematically investigated the recrystallization behavior, grain growth kinetics, hot deformation response and dynamic recrystallization mechanism of Co–Cr–W–Ni alloys [8,10,11,12,13,14,15,16,17]. Specifically, controlled static recrystallization (SRX) processing and oriented grain structure design have been proven to be effective strategies for optimizing the strength–ductility synergy of biomedical Co–Cr–W–Ni alloys [4,5,10,18,19,20,21,22,23]. The conclusions of these studies indicate that the recrystallization fraction, grain size distribution and residual deformation substructures are core microstructural parameters that determine the tensile mechanical properties of this alloy system. Furthermore, the precipitation of second-phase particles and carbides also plays a critical role in maintaining the microstructural stability of Co–Cr-based alloys during heat treatment. The carbon content of the alloy and the adopted annealing process parameters jointly affect the precipitation, dissolution and spatial distribution of carbides and other precipitated phases in Co–Cr and Co–Cr–W–Ni alloys [24,25,26,27,28]. Fine dispersed second-phase particles can inhibit grain boundary migration via the grain boundary pinning effect, which is generally interpreted based on the classical Zener pinning theory [29,30]. Accordingly, the structural stability of second-phase particles during the annealing process may exert a significant impact on the grain growth behavior and the final tensile properties of the alloy.

Electron backscatter diffraction (EBSD) is a powerful technique for characterizing recrystallization microstructures, grain boundary characteristics and deformation-induced substructures [31,32,33,34]. Kernel average misorientation (KAM) analysis is widely adopted to characterize local lattice curvature and local misorientation, which are closely correlated with the distribution of geometrically necessary dislocations and the magnitude of stored deformation energy, and is commonly used as a qualitative characterization indicator for the aforementioned structural features [32,33,34]. In addition, the dependence of alloy strength on grain size is conventionally described and analyzed via the classical Hall–Petch relationship [35,36,37]. Accordingly, the combination of EBSD-based quantitative microstructure characterization and systematic tensile testing constitutes an effective research approach for elucidating the inherent processing–microstructure–property correlation in cold-rolled L-605 alloy.

Existing research has systematically investigated static recrystallization, grain growth, bimodal grain structure formation, and thermomechanical processing of L-605 alloy and related Co–Cr–W–Ni alloys [8,18,19,20,21,22,23]. Therefore, this study does not present the above phenomena as newly discovered original results. The core contribution of this work is to conduct a systematic comparative analysis based on the same cold-rolled starting material, targeting three types of annealing processes: (i) recovery and partial to near-complete static recrystallization conducted at 800–950 °C; (ii) the thermal stability of fully recrystallized fine-grained microstructure under prolonged holding at 1000 °C; (iii) high-temperature grain growth behavior at 1200 °C. By comparing the variation trends of kernel average misorientation parameters and tensile properties under different conditions, the integrated processing–microstructure–property relationship of the alloy is established. This work was carried out to reveal the influences of annealing temperature and holding time on the microstructure and tensile properties of cold-rolled Co–20Cr–15W–10Ni alloy. The alloy was subjected to solution treatment, cold rolling and subsequent annealing under three sets of process conditions: annealing at 800–950 °C for 15 min was designed to investigate the microstructure evolution from partial recrystallization to near-complete recrystallization; isothermal holding at 1000 °C for 5–60 min was implemented to explore the thermal stability of the fully recrystallized fine-grained structure; annealing at 1200 °C for 5–60 min was conducted to characterize the high-temperature grain growth behavior. BSE imaging and electron backscatter diffraction technology (including inverse pole figure mapping and kernel average misorientation mapping) were employed to characterize the microstructural evolution of the alloy under different conditions, and corresponding tensile property tests were carried out to finally establish the processing–microstructure–property relationship of cold-rolled L-605 alloy.

## 2. Materials and Methods

The material used in this study was a Co–20Cr–15W–10Ni alloy (SamHwa steel Company, Busan, Republic of Korea). The chemical composition of the alloy was Co balance, 20.58 wt.% Cr, 14.90 wt.% W, 10.81 wt.% Ni, 2.67 wt.% Fe, 1.72 wt.% Mn, and 0.09 wt.% C. This composition corresponds to an L-605-type Co–Cr–W–Ni biomedical alloy investigated in previous studies [1,2,3,4,5,8]. The initial material was supplied as a bar with a diameter of 12 mm.

The as-received bar was subjected to solution treatment at 1200 °C for 30 min, followed by water quenching to room temperature. Subsequently, the solution-treated bar was processed via cold rolling at ambient temperature (25 °C), where the work roll diameter was 180 mm and the rolling speed was set as 1.0 m/min, giving a calculated rolling strain rate of 0.05 s^−1^. After 6 rolling passes, the final diameter of the cold-rolled bar was 9.3 mm. The total area reduction (denoted as RA) after cold rolling was 40%, which is calculated by the formula (RA = (A_0_ − A_f_)/A_0_ × 100), where A_0_ and A_f_ represent the initial and final cross-sectional areas of the bar, respectively. Three different annealing treatments were subsequently conducted on the cold-rolled specimens. In the first set of experiments, specimens were annealed at 800, 850, 900, and 950 °C for a holding time of 15 min, aiming to characterize the influence of annealing temperature on the recovery and static recrystallization (SRX) behavior. In the second set of experiments, specimens were annealed at 1000 °C with holding durations of 5, 15, 30, and 60 min to investigate the time-dependent evolution of the fully recrystallized fine-grained microstructure. In the third set of experiments, specimens were annealed at 1200 °C with holding durations of 5, 15, 30, and 60 min to examine the grain growth behavior of the recrystallized microstructure under high temperature. All annealing processes were terminated with water quenching to retain the high-temperature microstructure.

Microstructural observations were performed using backscattered electron (BSE) imaging and EBSD. BSE observations were conducted using a field-emission scanning electron microscope (FESEM: TESCAN MIRA III, Brno, Czech Republic) operated at 20 kV. EBSD analysis was performed using a field-emission scanning electron microscope (TESCAN Clara, Brno, Czech Republic) equipped with a TSL EBSD system operated at 20 kV. EBSD maps were acquired at a working distance of 15 mm with step sizes of 0.2 and 0.4 μm, depending on the grain size and observation area. Grain size measurements were carried out using OIM Analysis software (OIM V8). Grain boundaries were defined using a misorientation angle of 15°, which is commonly used to identify high-angle grain boundaries in EBSD-based recrystallization analysis [31]. KAM maps were used to qualitatively evaluate the distribution of local lattice distortion associated with deformation-induced substructures. Although KAM analysis does not directly provide the total dislocation density, it is widely used to assess local misorientation, lattice curvature, and geometrically necessary dislocation-related deformation structures in EBSD datasets [32,33,34].

Room-temperature tensile tests were conducted using cylindrical tensile specimens with a gauge length of 10 mm and a gauge diameter of 2.5 mm. Tensile tests were performed using an Instron 5982 testing machine (Instron, Norwood, MA, USA) at a constant strain rate of 1 × 10^−3^ s^−1^. Two specimens were tested for each condition, and the average values of ultimate tensile strength (UTS), yield strength (YS), and elongation were reported.

## 3. Results and Discussion

### 3.1. Microstructural Characterization

**Figure 1:**
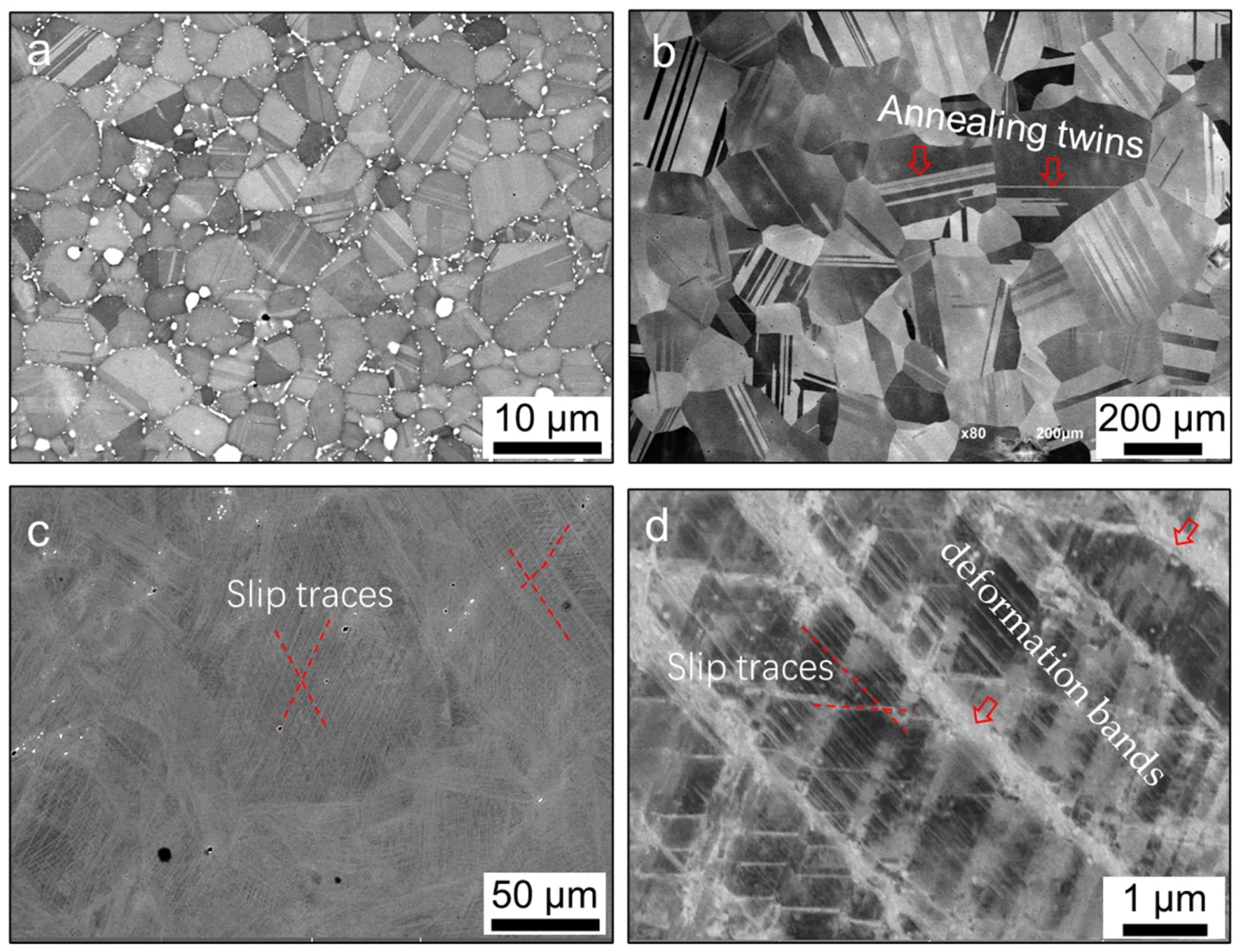
BSE images showing the microstructures of the initial (**a**), solution-treated (**b**), and cold-rolled (**c**,**d**) samples.

Figure 1 displays the microstructural characteristics of the initial, solution-treated and cold-rolled specimens in this study. The initial specimen presents a relatively fine-grained microstructure with a small quantity of second-phase particles (Figure 1a). Similar particle-containing microstructures have been reported in heat-treated Co–Cr–W–Ni alloys, which are generally related to carbide precipitation that depends on carbon content and heat-treatment parameters [24,25,26,27,28]. After solution treatment at 1200 °C for 30 min, the microstructure evolves into a coarse-grained structure with an average grain size of approximately 200 μm (Figure 1b). The number of second-phase particles observed in the initial specimen decreases significantly after solution treatment, indicating that most of the pre-existing second-phase particles are dissolved during high-temperature heat preservation. Meanwhile, annealing twins are detected in the solution-treated specimen, which is a typical microstructural feature of low stacking fault energy Co-based alloys after high-temperature annealing and grain growth [8,11,15]. After cold rolling, the alloy exhibits a typical deformed microstructure (Figure 1c). As shown in Figure 1c, the original grains are deformed, and deformation bands and slip traces can be clearly observed, while no deformation twins are formed in the alloy under this processing condition.

**Figure 2:**
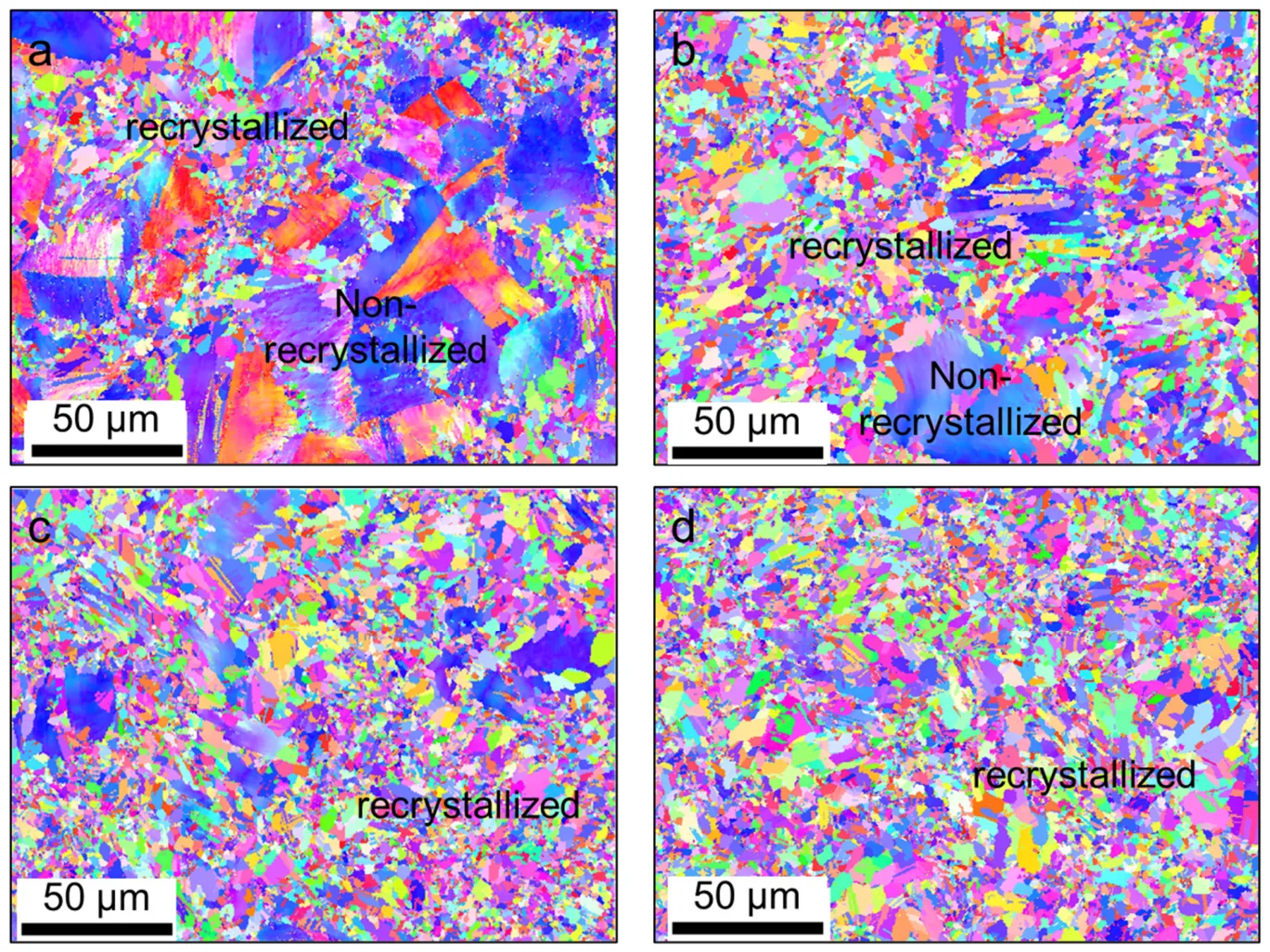
EBSD IPF maps showing the microstructures of the samples after the first-step annealing: (**a**) 800 °C, (**b**) 850 °C, (**c**) 900 °C, and (**d**) 950 °C.

Figure 2 presents the EBSD inverse pole figure maps of cold-rolled L-605 alloy after 15 min of annealing at 800–950 °C. The specimen annealed at 800 °C forms a partially recrystallized microstructure, which is composed of newly nucleated recrystallized grains and retained deformed/recovered regions (Figure 2a). The retained regions are characterized by relatively coarse and elongated morphology, confirming that the deformed initial microstructure has not been completely replaced by recrystallized grains at this annealing temperature. Such partial recrystallization behavior is consistent with existing reports on Co–Cr–W–Ni alloys after annealing or thermomechanical processing [4,8,18,19,20,21,22,23]. When the annealing temperature increases to 850 °C and 900 °C, the volume fraction of recrystallized grains rises gradually, and the proportion of retained deformed regions decreases significantly (Figure 2b,c). This phenomenon confirms that elevated annealing temperature promotes the nucleation and growth of recrystallized grains. Increasing temperature essentially enhances atomic mobility and grain boundary migration capability, thus accelerating the consumption of deformation-induced substructures. After annealing at 950 °C for 15 min, the alloy obtained a nearly fully recrystallized fine-grained structure with an average grain size of approximately 3.4 μm (Figure 2d), where the retained deformed regions were almost completely eliminated. This result indicates that 950 °C is sufficient to achieve near-complete static recrystallization under the cold rolling and annealing conditions adopted in this study. The microstructural transition from partial recrystallization at 800–900 °C to near-complete recrystallization at 950 °C is a key structural factor that dominates the tensile properties of the alloy, which will be discussed in subsequent sections.

**Figure 3:**
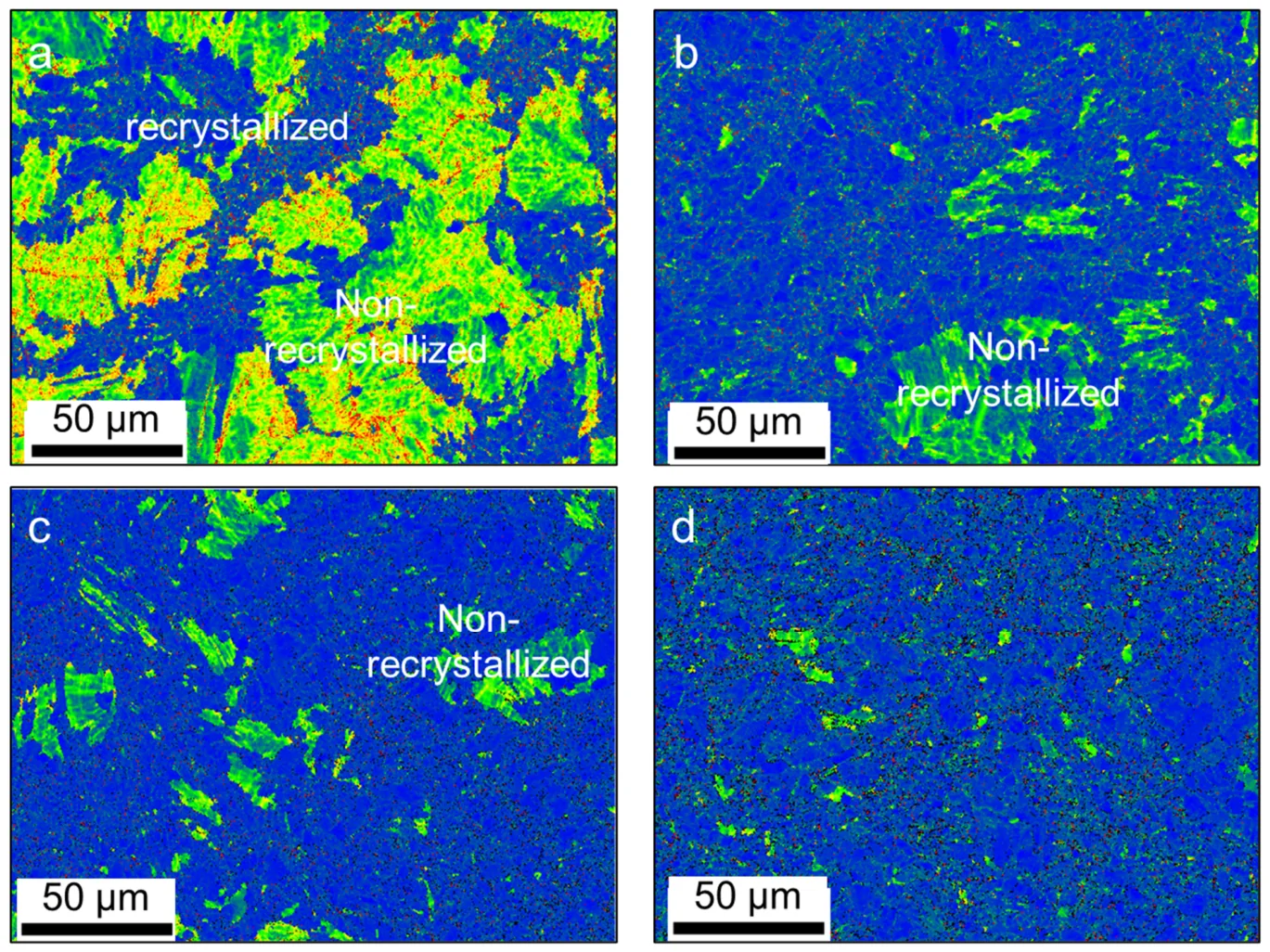
EBSD KAM maps showing the local lattice distortion of the samples after the first-step annealing: (**a**) 800 °C, (**b**) 850 °C, (**c**) 900 °C, and (**d**) 950 °C.

Figure 3 presents the EBSD kernel average misorientation (KAM) maps of the specimens annealed at 800–950 °C. In this study, KAM is employed as a qualitative indicator for characterizing local misorientation and lattice curvature induced by deformation-generated orientation gradients [32,33,34]. It should be noted that KAM cannot directly quantify the total dislocation density; thus, the KAM maps are interpreted to reflect the relative variation in local lattice distortion, rather than serving as a quantitative measurement of dislocation density. For the specimen annealed at 800 °C, a large proportion of high-KAM regions are observed, especially within the retained deformed and recovered microstructural zones (Figure 3a), which indicates that considerable local lattice distortion is still retained after annealing at this temperature. For the specimens annealed at 850 °C and 900 °C, the area fraction of high-KAM regions decreases gradually, demonstrating that recovery and recrystallization processes effectively eliminate the deformation-induced lattice distortion (Figure 3b,c). For the specimen annealed at 950 °C, the KAM value of most regions is at a low level, which is consistent with the formation of a nearly strain-free recrystallized microstructure (Figure 3d). The EBSD characterization results reveal that the microstructure evolves from a partially recrystallized state with significant deformation-induced orientation gradients to a more homogeneous, nearly fully recrystallized state with increasing annealing temperature in this range. Accordingly, the mechanical response of the alloy annealed at 800–950 °C is discussed based on the gradual elimination of deformation-induced strengthening effect and the formation of fine recrystallized grain structure, rather than estimating the total dislocation density via KAM results.

**Figure 4:**
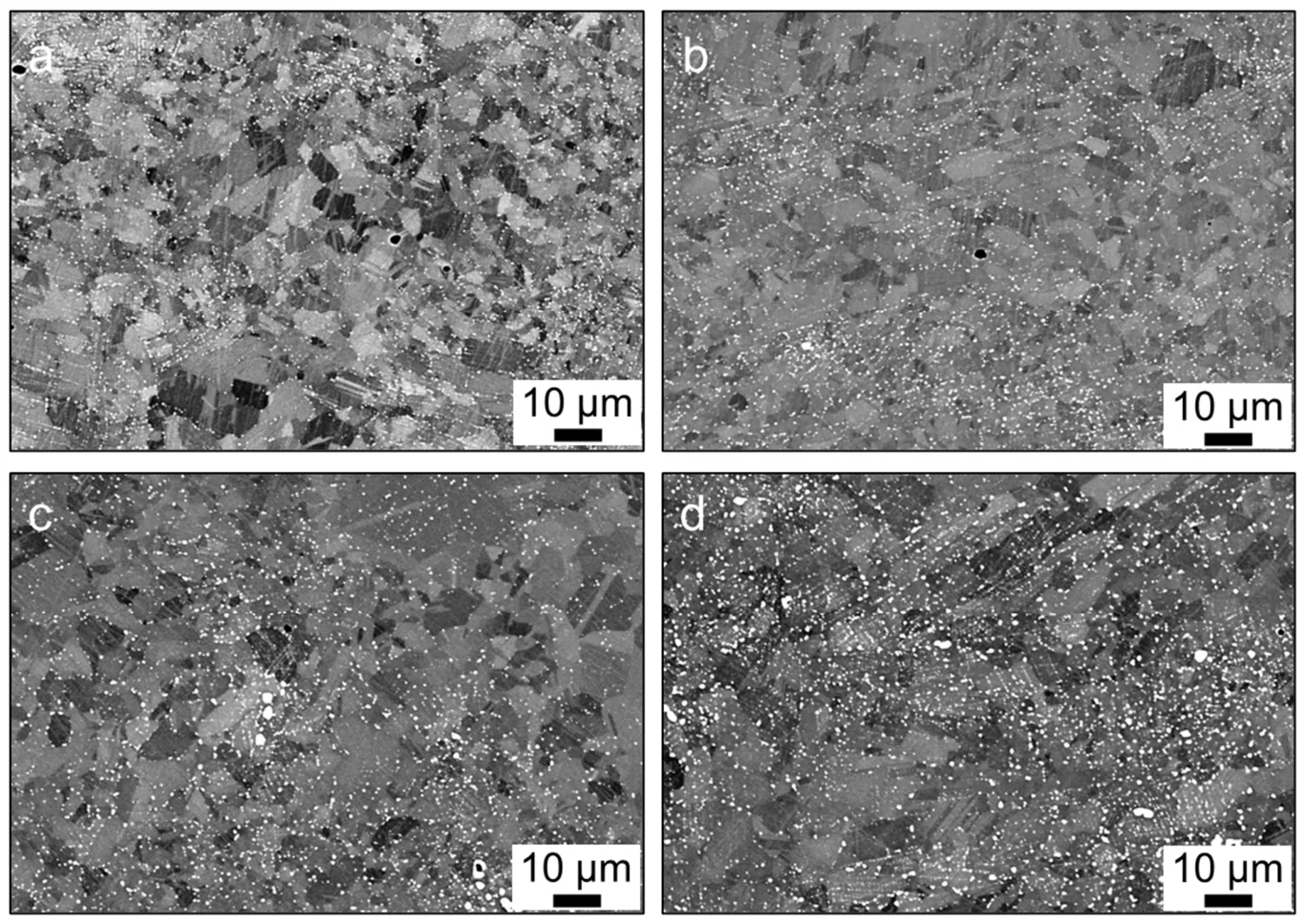
BSE images showing the microstructures of the samples after annealing at 1000 °C for (**a**) 5 min, (**b**) 15 min, (**c**) 30 min, and (**d**) 60 min.

Figure 4 presents BSE images of the specimens annealed at 1000 °C with holding durations ranging from 5 min to 60 min. All specimens exhibit complete recrystallized microstructures. Even after 60 min of annealing, the grain size remains relatively stable at approximately 5 μm, indicating that no noticeable grain coarsening occurs at 1000 °C under the experimental annealing conditions adopted in this work. Fine particles, which are observed as white dots in Figure 4, are detected in the BSE images of the specimens annealed at 1000 °C. According to previous studies [24,25,26,27,28], these particles are mainly assigned to carbides (M_23_C_6_). However, the BSE characterization alone cannot achieve accurate phase identification of the particles, since dedicated compositional and crystallographic characterization for these particles was not conducted in the present study. For this reason, they are referred to as fine second-phase particles in this paper, and their accurate chemical composition and crystal structure need to be verified in further research. The grain size stability at 1000 °C can be partially attributed to the existence of fine second-phase particles. Fine particles can inhibit grain boundary migration via the Zener pinning effect [29,30]. Therefore, the detected second-phase particles are likely responsible for the limited grain growth observed at 1000 °C. Nevertheless, this explanation remains at the qualitative stage, as the size, volume fraction, chemical composition and pinning force of the particles have not been quantitatively measured in this work.

The microstructural evolution behavior of the alloy at 1000 °C differs from that at 950 °C in two aspects. First, the alloy achieves complete recrystallization after short-time annealing at 1000 °C. Second, the grain size maintains a fine level of approximately 5 μm during prolonged holding. Thus, this condition is defined as a post-recrystallization fine-grain stability regime in this study. Particle-induced grain boundary pinning is a possible mechanism for this stability, but it is not considered as the only or experimentally confirmed mechanism.

**Figure 5:**
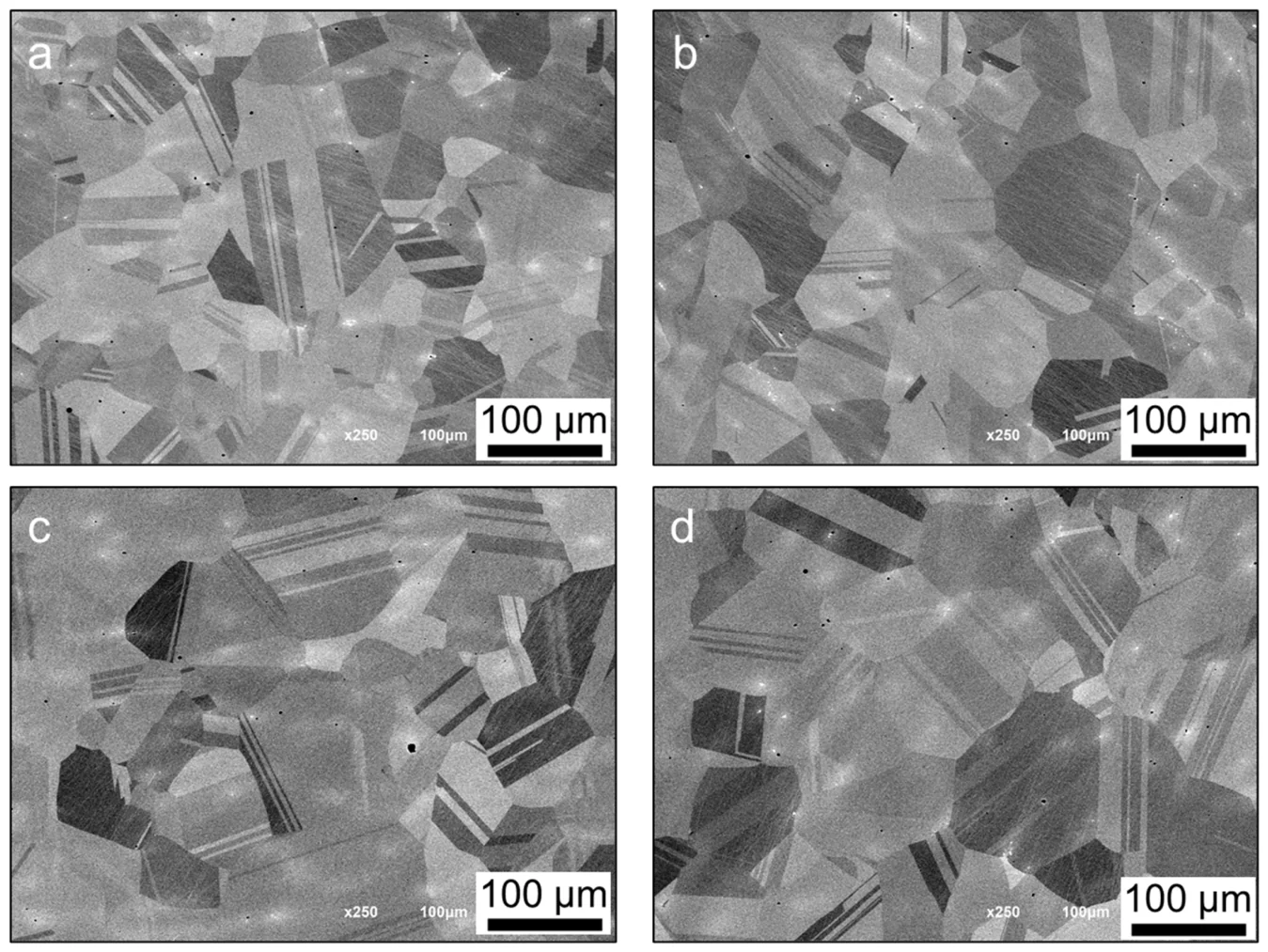
BSE images showing the microstructures of the samples after annealing at 1200 °C for (**a**) 5 min, (**b**) 15 min, (**c**) 30 min, and (**d**) 60 min.

Figure 5 presents BSE micrographs of the specimens annealed at 1200 °C with holding times ranging from 5 min to 60 min. Compared with specimens annealed at 1000 °C, those annealed at 1200 °C exhibited significant grain growth. The average grain size of the 1200-annealed specimens increased to approximately 90 μm, which is substantially larger than the average grain size of ~5 μm measured in specimens annealed at 1000 °C. At 1200 °C, fine second-phase particles were less clearly resolved in BSE micrographs than at 1000 °C. This observation can be attributed to one or more potential causes, including particle dissolution, particle coarsening, decreased particle volume fraction, or limitations of image contrast and resolution. The present experimental data do not allow for definitive differentiation among these possibilities. The significantly higher annealing temperature enhances atomic diffusion and grain boundary mobility, providing direct kinetic conditions for the observed substantial grain growth. A reduction in the effectiveness of Zener pinning from second-phase particles may also contribute to this behavior [29,30]. The microstructural differences between the 1000 °C and 1200 °C annealing conditions thus reflect a transition from a relatively stable fine-grained fully recrystallized state to a high-temperature grain growth regime. The substantial grain coarsening observed at 1200 °C is consistent with the increased grain boundary mobility at higher annealing temperatures. Reduced particle visibility or weakened particle pinning effects may also play a role, but without direct phase characterization or compositional analysis, particle dissolution cannot be confirmed as the governing mechanism.

In summary, three distinct microstructural regimes were obtained via annealing: (1) At temperatures between 800 °C and 900 °C, recovery and partial static recrystallization (SRX) resulted in mixed microstructures consisting of recrystallized grains and retained deformed/recovered regions. (2) At 950 °C, SRX was nearly complete, and a homogeneous fine-grained microstructure was formed. (3) At 1000 °C, the fully recrystallized fine-grained structure remained stable during holding times up to 60 min, which may be attributed to grain boundary pinning by second-phase particles. At 1200 °C, elevated grain boundary mobility induced significant grain growth, and the weakened pinning effect of second-phase particles may have further accelerated grain coarsening.

### 3.2. Mechanical Properties

**Figure 6:**
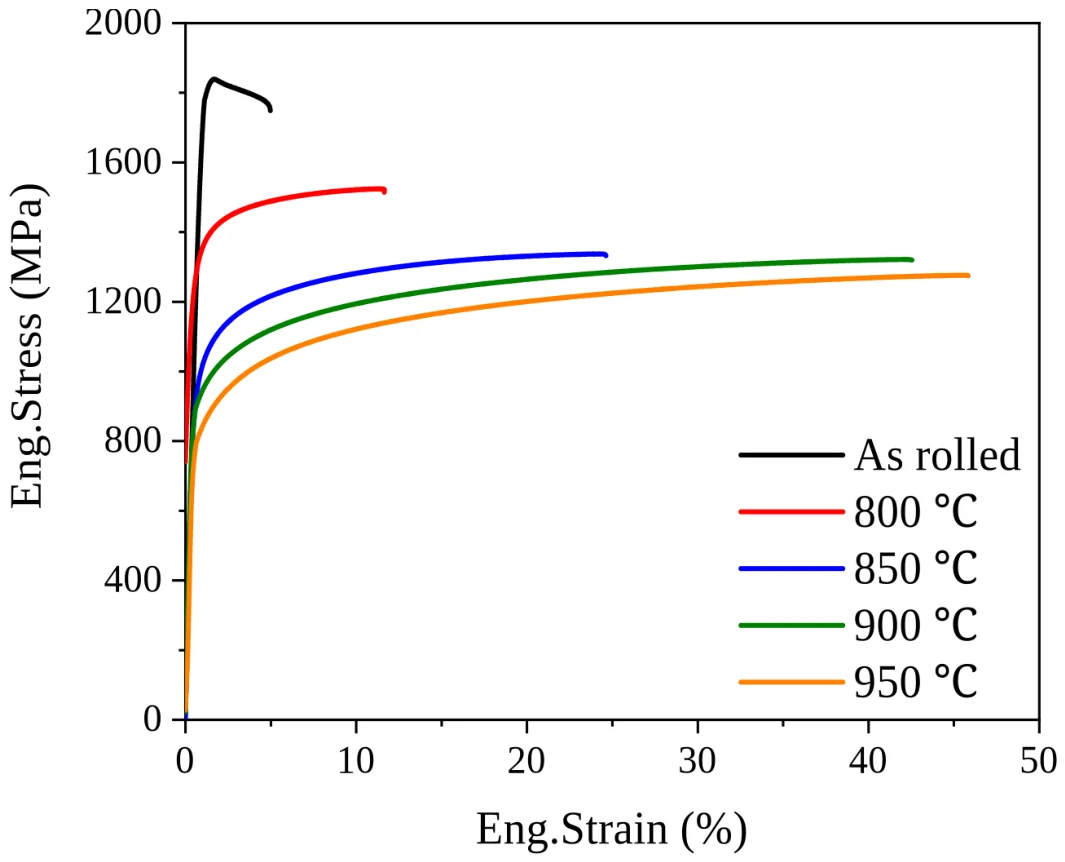
Engineering stress–strain curves of the cold-rolled L-605 alloy after annealing at 800–950 °C for 15 min.

Figure 6 present the tensile stress–strain curves of the specimens after 15 min of annealing at temperatures ranging from 800 to 950 °C. The corresponding tensile properties are shown in Table 1. Tensile properties vary significantly as a function of annealing temperature, which reflects that recrystallization and dislocation elimination exert a substantial influence on the macroscopic mechanical response of the material. The specimen annealed at 800 °C exhibits the highest strength, with a yield strength (YS) of approximately 1245 MPa and an ultimate tensile strength (UTS) of approximately 1550 MPa, while its elongation is restricted to approximately 12.6%. This mechanical behavior is consistent with the characteristics of the partially recrystallized microstructure and the existence of extensive high kernel average misorientation (KAM) regions, which indicate the retention of local lattice distortion and deformation-induced orientation gradients. Such retained deformation substructures correspond to a higher deformation-related strengthening contribution and reduce the material’s capacity for homogeneous plastic deformation. When the annealing temperature increases to 850 °C, the yield strength decreases to approximately 926 MPa, accompanied by a simultaneous increase in elongation. At annealing temperatures of 900 °C and 950 °C, the yield strength further decreases to approximately 883 MPa and 800 MPa, respectively, while the elongation increases to approximately 42.6% and 45.9%. The concurrent reduction in the area fraction of high-KAM regions and the increase in recrystallized fraction indicate that recovery and static recrystallization (SRX) proceed gradually with increasing temperature, and the contribution of retained deformation substructures decreases progressively.

Therefore, the decrease in strength from 800 °C to 950 °C is primarily attributed to the continuous elimination of deformation-related strengthening effects during recovery and SRX. Meanwhile, the fine recrystallized grains obtained after complete recrystallization provide grain boundary strengthening contribution, which conforms to the Hall–Petch strengthening mechanism [35,36,37]. Since neither the total dislocation density nor the Hall–Petch coefficient was measured in the present study, the relative contributions of different strengthening mechanisms are discussed qualitatively without quantitative decomposition. The improvement in ductility is associated with the gradual replacement of the heterogeneous deformed microstructure by recrystallized grains and the reduction in local lattice distortion. The predominantly low-KAM state of the specimen annealed at 950 °C indicates the formation of a more homogeneous recrystallized microstructure, which can accommodate plastic deformation more uniformly. Similar strength–ductility transition induced by recrystallization has been widely reported in existing studies on biomedical Co-Cr-W-Ni alloys [4,5,18,19,20,21,22,23].

**Figure 7:**
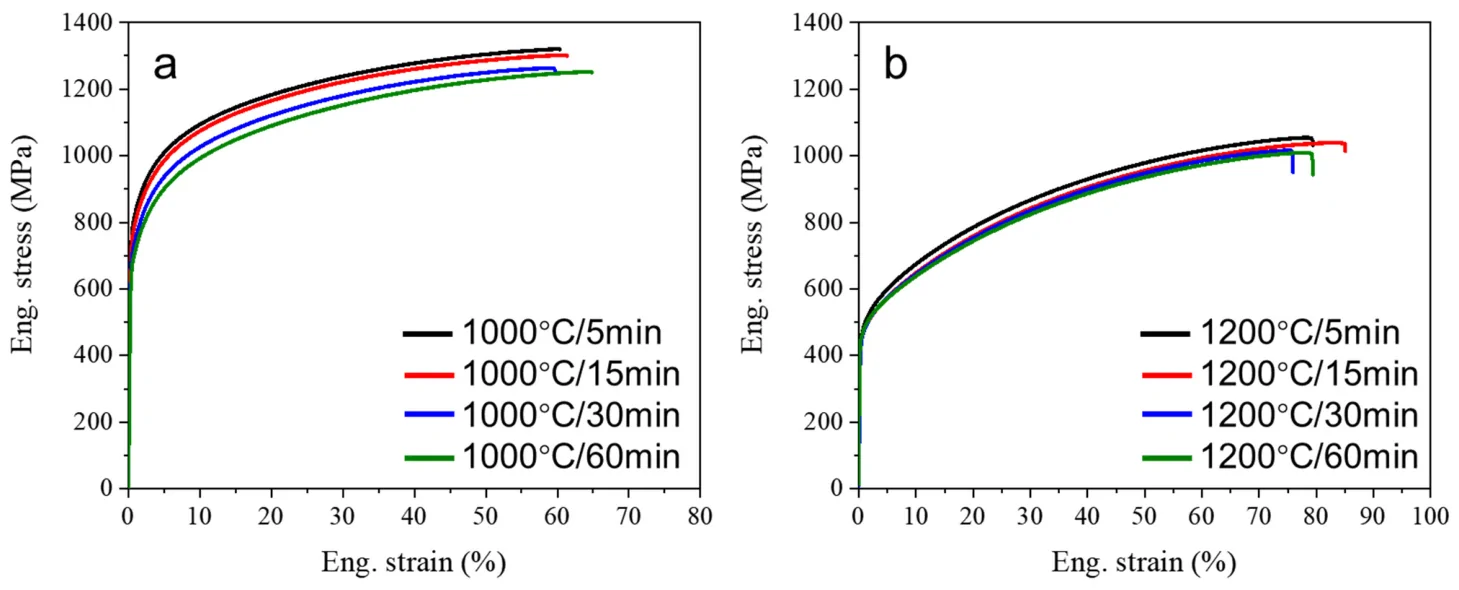
Engineering stress–strain curves of the cold-rolled L-605 alloy after annealing at high temperatures: (**a**) 1000 °C for 5–60 min and (**b**) 1200 °C for 5–60 min.

Figure 7 presents the tensile behavior of specimens annealed at 1000 °C and 1200 °C for holding durations of 5–60 min, and the corresponding tensile property parameters are summarized in Table 2. All specimens annealed at 1000 °C presented fully recrystallized microstructures and excellent tensile ductility. Compared with the partially recrystallized specimens obtained at lower annealing temperatures, the specimens annealed at 1000 °C exhibited lower yield strength (YS) but higher elongation. For the specimens annealed at 1000 °C, the ultimate tensile strength (UTS) decreased from approximately 1320 MPa after 5 min of annealing to approximately 1248 MPa after 60 min of annealing, while YS decreased from approximately 785 MPa to 668 MPa within the same holding time range.

These results demonstrate that extending the holding time at 1000 °C induced gradual softening of the material, even though no significant grain growth was detected. Since the average grain size was maintained at approximately 5 μm, the gradual strength reduction with increasing holding time at 1000 °C is not primarily caused by grain coarsening. Instead, this phenomenon may be attributed to the continuous relaxation of residual orientation gradients induced by plastic deformation, as well as the evolution of particle distribution and grain boundary states within the recrystallized microstructure. This interpretation is proposed qualitatively in the present work, as the total dislocation density and particle evolution were not quantitatively characterized.

The elongation of the 1000-annealed specimens remained at a high level, but did not show a monotonic trend with increasing annealing time. This non-monotonic variation may result from the combined effects of residual dislocation elimination, particle distribution adjustment, and microstructural homogenization. Considering that only two duplicate specimens were tested for each annealing condition, the observed elongation trend should be interpreted with caution in the absence of explicit characterization of data scatter. Overall, the experimental results indicate that annealing at 1000 °C achieves a superior combination of strength and ductility, compared with partially recrystallized microstructures obtained at lower annealing temperatures and coarse-grained microstructures formed at higher annealing temperatures.

The tensile response of specimens annealed at 1200 °C differed significantly from that of the 1000-annealed specimens. The 1200-annealed specimens exhibited lower strength but considerably higher elongation, which is consistent with the coarse fully recrystallized microstructure observed in Figure 5. For the 1200-annealed specimens, the UTS values were approximately 1052 MPa, 1031 MPa, 1002 MPa, and 1004 MPa after 5, 15, 30, and 60 min of annealing, respectively, and the corresponding YS values were approximately 455 MPa, 452 MPa, 442 MPa, and 448 MPa, respectively. The lower YS relative to the 1000 °C annealing condition is consistent with the characteristics of a coarse fully recrystallized microstructure. The reduction in grain boundary strengthening induced by grain coarsening, combined with the elimination of deformation-related strengthening after complete recrystallization, provides a reasonable explanation for the decreased YS. The elongation values of the 1200-annealed specimens were approximately 78.5%, 80.8%, 73.5%, and 78.4% after 5, 15, 30, and 60 min of annealing, respectively, which are considerably higher than those obtained after annealing at 800–950 °C and 1000 °C. The high elongation is attributed to the fully recrystallized and softened microstructure. However, elongation also did not present a simple monotonic dependence on annealing time, indicating that ductility is affected not only by grain size, but also by microstructural homogeneity, grain boundary character, residual second-phase particles, and experimental data scatter.

The comparative analysis of the microstructures and tensile properties obtained at 1000 °C and 1200 °C is of great significance for determining the reasonable processing window of L-605 alloy. Annealing at 1000 °C can obtain a fully recrystallized fine-grained microstructure with moderate-to-high strength and high ductility. In contrast, annealing at 1200 °C forms a coarse-grained microstructure with extremely high ductility but significantly reduced YS. Therefore, annealing at 1200 °C is applicable when maximum ductility or formability is required, but not suitable for application scenarios that require high YS retention.

The overall tensile behavior can be explained in terms of the sequential evolution of microstructural states, rather than as a quantitative superposition of individual strengthening mechanisms. At relatively low annealing temperatures, the retained deformation substructure is the dominant factor contributing to the high-strength, low-ductility state. With the progression of static recrystallization (SRX), the strengthening contribution of the retained deformation substructure gradually decreases, while ductility is concurrently improved. A fine recrystallized grain structure can still maintain a certain level of grain boundary strengthening contribution, while high-temperature grain growth will weaken this contribution.

After annealing at 800 °C, a large number of deformation-induced substructures remain in the alloy, which corresponds to the highest strength and the lowest elongation observed in the experiment. At 850 °C and 900 °C, recovery and partial SRX gradually reduce local lattice distortion and increase the recrystallization fraction, leading to a gradual decrease in strength and an increase in ductility. After annealing at 950 °C, near-complete SRX generates a fine-grained microstructure that is dominated by low KAM, with a significant improvement in ductility. At 1000 °C, the alloy achieves complete recrystallization and maintains a fine grain size of approximately 5 μm even when the annealing time is extended to 60 min. Under this condition, the ductility advantage of the recrystallized microstructure is combined with the residual grain boundary strengthening contribution provided by fine grains. Additionally, fine second-phase particles may inhibit grain boundary migration through Zener pinning [29,30], but the quantitative contribution of this effect has not been determined in the present study. At 1200 °C, significant grain growth occurs. Higher annealing temperatures enhance grain boundary mobility, and the reduced pinning effect of fine particles further decreases the resistance to grain boundary migration. The resulting coarse recrystallized microstructure exhibits extremely high elongation but a substantially lower yield strength (YS), which is consistent with the weakened grain boundary strengthening and the disappearance of retained deformation-related strengthening.

Based on the above analysis, the annealing behavior of the alloy can be divided into three distinct process regimes: partial SRX in the 800–900 °C range, near-complete to complete SRX with fine grain retention in the 950–1000 °C range, and high-temperature grain growth at 1200 °C. Among all the experimental conditions investigated, annealing in the 950–1000 °C range achieves the most optimal strength–ductility balance, while annealing at 1200 °C maximizes ductility at the cost of reduced yield strength. This interpretation is consistent with the conclusions of previous studies on SRX, grain structure design and thermomechanical processing of L-605 alloy [4,8,18,19,20,21,22,23].

Comparison with the results of representative existing studies on L-605 alloy supports the interpretation proposed in this paper. Li et al. [18] reported that for a 50% cold-rolled L-605 alloy annealed at 950–1100 °C for 15 min, SRX generates a bimodal grain structure, and a typical condition exhibits a yield strength of approximately 787 MPa, an ultimate tensile strength (UTS) of approximately 1278 MPa, and an elongation of approximately 53%. In a multi-pass thermomechanical processing route, Li et al. [21] achieved grain refinement to approximately 4 μm, with yield strength in the range of 593–738 MPa, ultimate tensile strength in the range of 1197–1304 MPa, and uniform elongation in the range of 54.7–61.1%. The 950–1000 °C annealing conditions in the present study fall into the same fine-grained, high-ductility processing category as reported in the above studies, while the 1200 °C condition enters a coarse-grained regime with significantly lower yield strength. Due to the differences in elongation definition and specimen geometry among different studies, the performance values cited above are only used for contextual comparison, not for strict one-to-one performance ranking.

## 4. Conclusions

The effects of annealing temperature and time on the microstructure and tensile properties of cold-rolled L-605 Co–Cr–W–Ni alloy were investigated. The main conclusions are summarized as follows:

1. Annealing at 800–900 °C for 15 min obtained partially recrystallized microstructures consisting of new recrystallized grains and retained recovered/deformed regions. Annealing at 950 °C achieved nearly complete recrystallized fine-grained structure, with an average grain size of ~3.4 μm. EBSD KAM analysis showed local misorientation and lattice distortion decreased gradually as annealing temperature rose from 800 °C to 950 °C, which matches the progressive development of recovery and SRX. KAM was not used as a direct indicator of total dislocation density. Annealing at 1000 °C for 5–60 min obtained fully recrystallized microstructures, with average grain size stable at ~5 μm. Fine second-phase particles may restrict grain boundary migration, but their phase composition and quantitative pinning effect were not determined. Annealing at 1200 °C for 5–60 min caused significant grain growth to ~90 μm. The coarsening is attributed to enhanced grain boundary mobility at higher temperature; reduced effective particle pinning may contribute additionally, but particle dissolution was not directly verified.
2. As the annealing temperature increased from 800 °C to 950 °C, the tensile strength of the experimental alloy decreased gradually, while the elongation exhibited an increasing trend. Specifically, the yield strength (YS) decreased from approximately 1245 MPa at 800 °C to approximately 800 MPa at 950 °C, and the elongation increased from 12.6% to 45.9%. This mechanical property evolution is consistent with the gradual elimination of deformation-induced strengthening effects during the recovery and static recrystallization (SRX) processes, as well as the formation of a more homogeneous recrystallized microstructure. When annealed at 1000 °C, the YS decreased from approximately 785 MPa after 5 min of holding to approximately 668 MPa after 60 min of holding, while high ductility was still maintained. Given that significant grain growth was restricted at this temperature, the gradual softening of the alloy can be qualitatively attributed to the continuous relaxation of residual deformation-induced orientation gradients, as well as potential changes in the state of second-phase particles and grain boundaries. When annealed at 1200 °C, the alloy exhibited a YS in the range of approximately 442–455 MPa and an elongation in the range of approximately 73.5–80.8%. This low strength and high ductility characteristic matches well with the coarse fully recrystallized microstructure obtained at this temperature: in this microstructure, deformation-related strengthening effects have been completely eliminated, and the grain boundary strengthening contribution is reduced due to grain coarsening.
3. The strength–ductility balance is therefore associated with the evolution from retained deformation substructures to a fine recrystallized grain structure and, at higher temperature, to a coarse recrystallized structure. Annealing near 950–1000 °C provides a balanced combination of strength and ductility under the present processing conditions, whereas annealing at 1200 °C provides very high ductility at the expense of YS.

## Figures and Tables

**Table 1:** Tensile properties of the cold-rolled L-605 alloy after annealing at 800–950 °C for 15 min.

Annealing Temperature	UTS (MPa)	YS (MPa)	EL (%)
800 °C	1550	1245	12.6
850 °C	1344	926	26.1
900 °C	1322	883	42.6
950 °C	1276	800	45.9

**Table 2:** Tensile properties of the cold-rolled L-605 alloy after annealing at 1000 and 1200 °C for different holding times.

Annealing Time	1000 °C			1200 °C		
	UTS (MPa)	YS (MPa)	EL (%)	UTS (MPa)	YS (MPa)	EL (%)
5 min	1320	785	60.3	1052	455	78.5
15 min	1302	765	59.5	1031	452	80.8
30 min	1252	698	56.7	1002	442	73.5
60 min	1248	668	63.6	1004	448	78.4

## Data Availability

The original contributions presented in this study are included in the article. Further inquiries can be directed to the corresponding author.
